# Cervical anticancer activities of *Annona squamosa* Linn. leaf isolate

**DOI:** 10.14202/vetworld.2022.124-131

**Published:** 2022-01-24

**Authors:** Made Dira Swantara, Wiwik Susanah Rita, Made Asmarani Dira, Kadek Karang Agustina

**Affiliations:** 1 Department of Applied Chemistry, Faculty of Mathematics and Natural Sciences, Udayana University, Denpasar, Bali 80225 Indonesia; 2 Clinical and Community Pharmacy Study Program, Faculty of Health, Bali Institute of Technology and Health, Denpasar, Bali 80225, Indonesia; 3 Department of Public Health, The Faculty of Veterinary Medicine, Udayana University, Denpasar, Bali 80225, Indonesia.

**Keywords:** *Annona squamosa* Linn, cervical anticancer activities, HeLa cells

## Abstract

**Background and Aim:**

Cancer is one of the leading causes of death, the need for new anticancer herbal drugs is becoming more urgent considering the side effects of synthetic drugs. This study aimed to determine the anticancer activity of isolates derived from the methanol extract of *Annona squamosa* Linn. leaves and to identify the compounds that have an active effect against HeLa cells.

**Materials and Methods:**

The leaf metabolites of *A. squamosa* L. were extracted using methanol at room temperature (28°C) and were partitioned into n-hexane, chloroform, and n-butanol. The toxicity test of these extracts was conducted using a brine shrimp lethality assay. Furthermore, the most toxic extracts were separated and purified using silica gel column chromatography to yield four isolate fractions: FA, FB, FC, and FD. The most toxic isolates were tested for anticancer against HeLa cells, and their compounds were identified using liquid chromatography-mass spectrometry.

**Results:**

The results showed that the most toxic isolate with an LC_50_ value of 100.00 ppm had a potency similar to that of an anticancer agent with an IC_50_ value of 70.9021 ppm. Furthermore, the five compounds identified in this isolate include (6S, 7aR)-6-hydroxy-4,4,7a-trimethyl-6,7-dihydro-5H-1-benzofuran-2-one or loliolide, cocamidopropyl betaine, N-[3-(dimethylamino)propyl]dodecanamide or lauramidopropyl dimethylamine, linolenic acid, and 1-dodecyl-2-azepanone or laurocapram.

**Conclusion:**

It can be concluded that the leaf isolates of *A. squamosa* Linn. had shown anticancer activities against cervical cancer.

## Introduction

The need for new anticancer drugs is becoming more urgent since the ones presently being used are expensive and have low selectivity [1]. Globally, cancer is one of the leading causes of death, considering that the anticancer drugs that have been developed have not yet produced satisfactory results without adverse side effects [2]. Therefore, the search for new sources to produce anticancer compounds is continuously carried out by exploring biological materials (organisms) on land and sea [3]. These terrestrial and marine organisms are the oldest materials used to maintain health or treat diseases [4]. Although these biological organisms have been widely used, scientific data on their efficacy is insufficient [5]. At present, biological organisms as a source of traditional medicine are an important object of study in the development of pharmaceutical science [6]. One of the plants that are traditionally used as medicinal ingredients is the *Annona squamosa* Linn. plant [7]. This plant is used to prevent and treat heart diseases, flu, cough, acute dysentery, diarrhea, cancer, asthma, constipation, diabetes, hypertension, fever, and skin diseases and also has anti-inflammatory properties [8-12].

Several studies on the anticancer activities of *A. squamosa* L. plants have been conducted [13-26]. However, these studies have not reported any cervical anticancer activity. Similarly, the two studies on the cervical anticancer activities of *A. squamosa* L. conducted by El-Darier and Abdelhady [27] used fruit seeds and fruit pulps [28], respectively.

A preliminary study on anticancer agents was conducted through a toxicity test using *Artemia salina* L. larvae [29,30]. A previous study also stated that when the level of toxicity (LC_50_) was <1000 ppm, the material can potentially become an anticancer agent [31-33]. Meanwhile, the cervical anticancer assay is conducted using HeLa cells because cell lines were derived from cells that grow as semi-adherent cells. These HeLa cells were derived from human cervical cancer epithelial cells, which were isolated in 1951 from the uterus of a woman with cervical cancer named Henrietta Lacks [34,35].

The objectives of this study were to test the anti-cervical cancer activity of the leaf isolates of *A. squamosa* L. and identify compounds that are toxic to cancer cells.

## Materials and Methods

### Ethical approval

This study was not conducted on living humans or animals so, ethical approval was not necessary

### Study period and location

*A. squamosa* L. leaf study was conducted in two phases, the first phase from September to December 2019 to determine the toxicity of the extract only. The second phase was conducted from June to October 2021 where the leaf sample used was the same plant as the previous research. The study was conducted in the Laboratory of Chemistry, Udayana University, Bali, Forensic Laboratory of Indonesian Police, and Laboratory of Primates, IPB University Bogor.

### Materials

*A. squamosa* L. leaves were harvested from healthy trees on the 5^th^ to 10^th^ leaves from Buleleng and Karangasem regencies, Bali. The leaves of *A. squamosa* Linn. were identified by I Made Sumerta and I Nyoman Sudiatna in the Indonesian Institute of Science before being used. Furthermore, the chemicals used were methanol, n-hexane, chloroform, n-butanol, and other solvents with pro analysis quality, produced by Merk, Germany. Brine shrimp *A. salina* eggs were purchased from American Technology, while the cell line was purchased from the Primate Study Centre, Bogor Agriculture University. Moreover, the liquid chromatography-mass spectrometry (LC-MS) was conducted using an ACQUITY Ultra Performance Liquid Chromatography (UPLC)®H-Class System [36].

### Sample preparation

Approximately 5 kg of fresh samples were cleaned and cut into smaller pieces, which were dried to produce 2750 g of clean and dry samples. These samples were ground and sieved to produce 2470 g of sample powder.

### Water content determination

The determination of water content was conducted using the thermogravimetric method [37]. Furthermore, the clean empty porcelain plates were dried in an oven at 105°C for 30 min, cooled in a desiccator for 15 min and weighed, and were further heated to dryness at 105°C. This procedure was repeated until the plate weight was constant (W_0_). A total of 2 g of sample powder was put into the plate and weighed (W_1_). The plate which contained the sample was dried in an oven at 105°C for 3 h and cooled in a desiccator for 15-30 min. The plates and the contents were weighed and dried again for 1 h, cooled in a desiccator, and weighed until a constant weight (W2) was achieved. Water content was determined using the equation below:


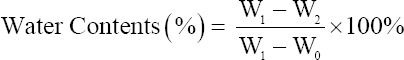


### Extraction of secondary metabolites

The fine and dry samples were extracted by maceration using methanol for 24 h. After 24 h, the extract was filtered, and the dregs were further extracted using fresh methanol, and the process was repeated 3 times. All methanol extracts were collected and evaporated using a vacuum rotary evaporator at 40°C to obtain the crude extract of methanol [38,39]. The crude methanol extract was tested for toxicity using *A. salina* Leach larvae, followed by a partitioning process. Approximately 1 kg of sample powder was extracted with 3×2.5 L of methanol to produce 5 L of extract. All methanol extracts were evaporated to produce 104 g of crude methanol extract. Approximately 50 g of crude extract was partitioned, each with 5×100 mL n-hexane, chloroform, and n-butanol yielding EH, EC, and EB (14.3, 2.57, and 9.05 g, respectively).

### Partition

This crude extract was partitioned into three solvents, namely, n-hexane, chloroform, and n-butanol, to obtain the n-hexane extract (EH), chloroform extract (EC), and n-butanol extract (EB). These three extracts (EH, EC, and EB) were tested for toxicity, and the most toxic extracts were separated and purified.

### Separation and purification

The most toxic extracts were separated by silica gel column chromatography using suitable eluents to obtain several fractions which were tested for toxicity. Furthermore, a purity test was conducted on the most toxic fraction by thin-layer chromatography (TLC) using several eluent systems. When the isolates provided a single spot on the TLC plate with various eluent systems, it was purely based on the TLC. Therefore, an anticancer test was conducted for the isolates on the HeLa cells [38,39].

### Toxicity test

The medium for larvae hatching was prepared by filtering seawater, placed in an aquarium and divided into dark and bright parts. *A. salina* eggs (50 mg) were placed or immersed in the dark part and left for 48 h until mature larvae were hatched and ready for testing. Twenty milligrams of methanol extract were dissolved into 2 mL of solvent, and the solutions had volumes of 500, 50, and 5 mL each. Furthermore, each solution was inserted into the test tube, and the solvent was evaporated. Dimethyl sulfoxide (nearly 50 mL), seawater (1 mL), and 10 larvae were placed into a test tube that contained the sample. The solvent was later evaporated, and seawater was added to a volume of 5 mL to obtain extract concentrations of 1000, 100, and 10 ppm [40]. A concentration of 0 ppm (solution without the addition of the extract) was prepared as a control, and the death of *A. salina* larvae was measured after 24 h [31]. Meanwhile, the standard assessment of larval mortality was performed when the larvae showed no movement during an observation period of several seconds [41]. The number of live and dead larvae was recorded and the data were analyzed to determine the LC_50_.

### Anticancer test

The toxic isolate was assayed for its anticancer activity against HeLa cells [42], which were cultured in a Roswell Park Memorial Institute 1640 medium. The initial number of cells was counted using a microscope. The cells were trypsinized, harvested, and centrifuged to form two layers, namely, sediment and supernatant. Meanwhile, the supernatant was removed and the precipitate was pelletized, which was followed by the addition of 1 mL of complete medium, and the number of cells was counted using a neubauer counting chamber (Hecht Glaswarenfabrik GmbH & Co KG, Germany). Subsequently, 2×10^4^ cells were seeded in 100 mL of the medium in a 96-well plate and incubated for 1-2 h for the cells to adhere. Subsequently, 100 mL extracts of the test material were added at various concentrations (1000, 500, 250, 125, 62.5, 31.25, 15.62, 7.81, 3.91, 1.95, 0.97, 0.48, 0.24, 0.12, and 0.06 mg/mL) to make a total volume of 200 mL in each well. The cells were incubated for 24 h at 37°C and were observed using the microscope after incubation. Furthermore, 3-(4,5-dimethylthiazole-2-yl)-2,5-diphenyltetrazolium bromide (MTT; 5 mg/mL) was added to each well and incubated for 4 h. Subsequently, a 10% stop solution of sodium dodecyl sulfate (SDS) in 0.01 N HCl was added to each well and incubated overnight. The absorbance at 500 nm was observed using an enzyme-linked immunosorbent assay plate reader [43].

### Compound identification

The most toxic isolates were identified by their compounds using LC-MS/MS with the following working parameters:

(1)Chromatographic Separation LC System: UPLC
(2)Column: C18 (1.8 mm 2.1×100 mm) HSS
(3)Temperature: 50°C (Column) and 25°C (room)
(4) Mobile phase: Water+5 mM ammonium formic acid (A) and acetonitrile+0.05 % formic acid (B)
(5) Flow rate: 0.2 mL/min (step gradient) running 23 min (see slide moving phase)
(6) Injection volume: 5 mL (filter through 0.2 mm syringe filter first)
(7) Mass spectrometry system: Electrospray Ionization
(8)Mode: Positive mode
(9)Mass analysis range: 50-1200 m/z
(10)Source temperature: 100°C
(11)Desolvation temperature: 350°C
(12)Cone gas flow: 0 L/h
(13)Desolvation gas flow: 793 L/h
(14)Collision energy: 4 volt (1)
(15)Ramp collision energy: 25-60 volt (2).

Moreover, the samples were prepared using the solid-phase extraction method, which was a chromatographic separation of the solid phase. This was carried out using the Hydrophilic-Lipophilic-Balanced stationary phase (the Waters brand), made of N-vinyl pyrrolidone for hydrophilic and divinyl for lipophilic. Furthermore, methanol was used for the mobile phase eluting polar compounds, while dichloromethane was used for eluting non-polar compounds. The sample that has been diluted in methanol was put into the Water’s Column Package in the form of a syringe that contained the stationary phase eluted with methanol. The sample was eluted with dichloromethane in the same column and accommodated [36]. The elution results were inserted into the LC-MS/MS tool for analysis. The chromatogram obtained was identified using theMassLynx V4.1 program (Waters Corp., MA, USA) to determine its mass spectrum.

## Results

### Water content determination

The measurement of water content in the sample was performed in the early stages of the study on natural materials. Meanwhile, high water content makes the sample easily contaminated with fungi and prevents solvents from entering the cells. This also causes imperfect extraction results. Moreover, the water content of the sample powder in this study was 8.13±0.1%.

### Extraction of secondary metabolites

Table-1 shows the results of the methanol crude extract toxicity test. Table-2 shows the results of the toxicity tests of the three extracts. Based on the data in Table-2, the EC was the most toxic extract with an LC_50_ value of 52.48 ppm.

**Table-1:** Toxicity of the leaf methanol crude extract of *Annona squamosa.*

Sample	Mortality			LC_50_ (ppm)

	10 ppm	100 ppm	1000 ppm	
Methanol extract	17.73±7.51	92.09±7.70	100±0	26.30

EH=n-hexane extract, EC=Chloroform extract, EB=n-butanol extract

**Table-2:** Toxicity of EH, EC, and EB.

Extracts	Mortality			LC_50_ (ppm)

	10 ppm	100 ppm	1000 ppm	
EH	16.69±3.40	48.38±1.07	93.99±0.21	104.71
EC	19.58±0.72	61.91±4.13	94.64±0.17	52.48
EB	3.34±2.89	20.94±3.70	3.34±2.89	323.59

EH=n-hexane extract, EC=Chloroform extract, EB=n-butanol extract

### Separation and purification

The EC was separated and purified using silica gel column chromatography with an eluent of n-hexane: chloroform (1:4) and gave four fractions (fraction A [FA], fraction B [FB], fraction C [FC], and fraction D [FD]). These fractions were tested for toxicity, and the results are shown in Table-3. Based on Table-3, FA with an LC50 value of 100.00 ppm was tested for purity using the silica gel TLC method with various eluent systems. The results showed that the FA test has one spot on all eluent systems and the FA was declared pure by TLC.

**Table-3:** Toxicity values of FA, FB, FC, and FD.

Fractions	Mortality			LC_50_ (ppm)

	10 ppm	100 ppm	1000 ppm	
A	17.36±1.20	50.16±6.04	90.30±3.58	100.00
B	10.17±0.30	36.90±1.03	82.22±3.85	204.17
C	22.55±4.24	59.53±8.25	94.53±0.36	316.22
D	4.61±0.12	20.47±0.82	71.79±4.43	407.38

FA=Fraction A, FB=Fraction B, FC=Fraction C, FD=Fraction D

### Anticancer activity assay

Anticancer activity against HeLa cells was determined using the MTT assay colorimetric method to measure cell proliferation [40]. The principle of this assay is the reduction of yellow tetrazolium salt MTT to purple formazan crystals by living mitochondria [44]. In this process, MTT is absorbed into live cells and reduced by succinate dehydrogenase in the electron transport chain of the mitochondria to formazan. The formazan crystals produced are dissolved in 10% SDS to form a purple solution. The MTT reduction occurs only in living cells through the nicotinamide adenine dinucleotide hydrogen and nicotinamide adenine dinucleotide phosphate cofactor pyridine nucleotides. Hence, the amount of formazan formed is proportional to the number of live cells [45]. Furthermore, the optical density (OD) of each well was measured at 595 nm using a microplate reader. All tests were conducted in triplicate and the average OD value was converted to a percentage inhibition. Table-4 shows the results of the absorbance observations and percentage growth inhibition of the HeLa cells after being given *A. squamosa* L. isolates (FA).

**Table-4:** Toxic isolate (Fraction A) inhibition.

Concentration (ppm)	Optical Density			% Inhibition			
	
	1	2	3	1	2	3	Average
100	0.115	0.114	0.116	59.507	59.859	59.155	59.507±0.352^a^
50	0.154	0.153	0.153	45.775	46.127	46.127	46.010±0.203^b^
25	0.167	0.169	0.167	41.197	40.493	41.197	40.962±0.406^c^
12.5	0.178	0.177	0.177	37.324	37.676	37.676	37.559±0.203^d^
6.25	0.192	0.194	0.192	32.394	31.690	32.394	32.159±0.406^e^
3.125	0201	0.200	0.200	29.225	29.577	29.577	29.460±0.203^f^
1.65	0.202	0.203	0.202	28.873	28.521	28.873	28.756±0.203^f^
0.78	0.219	0.218	0.219	22.887	23.239	22.887	23.004±0.203^g^
0.39	0.226	0.225	0.226	20.422	20.775	20.422	20.540±0.204^h^
0.195	0.239	0.237	0.238	15.845	16.549	18.197	16.864±1.207^i^
Cell Control	0.284	0.284	0.284	0	0	0	0

a-iValues followed by the same letters in the same column are not significantly different according to Duncan’s Multiple Range Test at p *<*5%.

**Figure-1:**
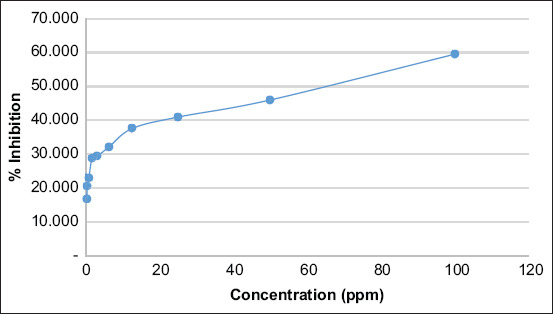
The curve correlation between sample concentration and inhibition. From Figure-1, the equation of the graph was y=24.5923+5.9624. ln x, with coefficient determination R^2^=0.9370. The IC_50_ was calculated using the equation below. 50=24.5923+5.9624 ln x ln x= (50–24.5923)/5.9624=4.2613 x=70.9021

The regression line equation model y=a+b ln x was constructed using the data in Table-4, and the value obtained was y=24.5923+5.9624 ln x (R^2^=0.9370). Figure-1 shows the graph of the relationship between the sample concentration and percentage growth inhibition for the determination of the IC_50_ value.

The IC_50_ toxic isolate from the methanol extract of *A. squamosa* L. was 70.9021 ppm. Based on the US National Cancer Institute, cytotoxic compounds are classified as (1) very toxic with a IC_50_ value of ≤20 mg/mL, (2) moderately cytotoxic or quite active when the IC_50_ value is within 21-200 mg/mL, and (3) weak cytotoxic when the IC_50_ value is within 201-500 mg/mL or ≥500 mg/mL. They are also classified into non-toxic categories [46].

### Compound identification

**Figure-2:**
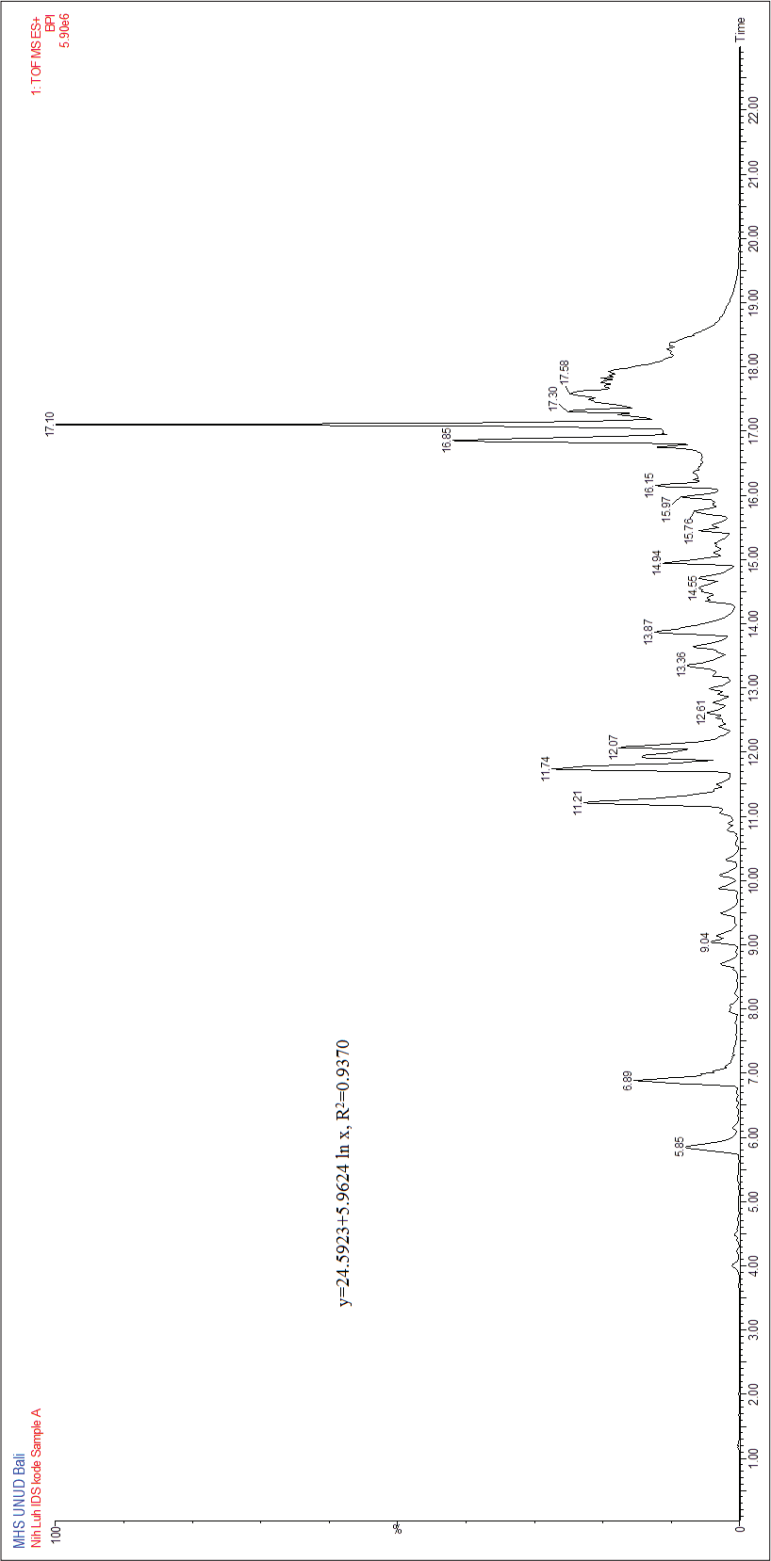
Liquid chromatography with tandem mass spectrometry chromatogram of fraction A.

Figure-2 shows the results of the identification of *A. squamosa* L. using the LC-MS/MS instrument, which indicated the presence of 16 peaks with similar retention times (Rt) in low- and high-energy chromatograms. Meanwhile, 11 peaks were assumed to be noise peaks because the base peaks in the low- and high-energy spectra were not similar. Therefore, only five peaks were established, namely, Rt 5.851, 5.851, 10.329, 13.213, and 15.448 min which were used to identify the compounds present in the sample. Based on the LC chromatogram, the number of peaks indicated five compounds in the isolate. These compounds were identified using mass spectra, and the results are shown in Table-5.

**Table-5:** Compounds identified in the isolate of the A fraction of *Annona squamosa* leaves.

Peak	Retention time (min)	M^+^ion (m/z)	Molecular formula	Compounds name	Compounds group
1	5.851	197.1183	C_11_H_16_O_3_	(6*S*,7*aR*)-6-hydroxy-4,4,7*a*- trimethyl-6,7-dihydro-5*H*-1-benzofuran-2-one or Loliolide	Monoterpenoids
2	9.894	343.2959	C_19_H_38_N_2_O_3_	Cocamidopropyl betaine	Amine
3	10.329	285.2905	C_17_H_36_N_2_O	N-[3-(Dimethylamino) propyl] dodecanamide or Lauramidopropyl dimethylamine	Amine
4	13.213	279.2313	C_18_H_30_O_2_	Linolenic acid	Fatty acid
5	15.448	282.2809	C_18_H_35_NO	1-Dodecyl-2-azepanon or Laurocapram	Alkaloids

## Discussion

The water content test was determined based on the evaporation process of the water contained in the sample by comparing the mass of the evaporated water content with the mass of the whole sample. Meanwhile, a material achieves optimal stability and avoids microbial growth when it has low water content [47]. When the solvent cannot be mixed with water, the high water content hinders the solvent from entering the cell to hamper the extraction process. The extraction process occurs quickly in the sample at a maximum water content of 10% [47,48]. In this study, the water content of the sample was 8.13±0.1%, which indicated that the sample is eligible for extraction. Methanol was used in the maceration process due to its ability to dissolve almost all organic compounds from non-polar to polar, which effectively extracted secondary metabolites. This was because methanol has polar (-OH) and non-polar groups (-CH_3_) that were close together to attract polar and non-polar compounds [49].

Based on the toxicity test, the crude extract of methanol, partitioned EC, and isolate *A. squamosa* L. from the separation had LC_50_ values of 26.30, 52.48, and 100.00 ppm, respectively. This showed that the toxic compounds in the leaf extract of *A. squamosa* L. were synergistic.

The anticancer activity (IC_50_) of *A. squamosa* L. leaf isolate against HeLa cells was 70.9021 ppm, which belonged to the moderate cytotoxic category [46]. This result is lower than that of the study of Mehta and Paliwal [28] who obtained an inhibition of 67.15% (for HeLa cells) at a sample concentration of 100 ppm because the same sample concentration gave an inhibition of 59.507%. Similarly, when compared with the results of the study by El-Darier and Abdelhady [27], the *A. squamosa* L. fruit pulp extract exhibited an anticancer activity against HeLa cells with an IC_50_ value of 38.09 ppm. In a previous study by Yajid *et al*. [50], the fruit pulp of *A. muricata* also exhibited an anticancer activity against HeLa cells with an IC_50_ value of 32.89 ppm. Furthermore, Widyanto *et al*. [51] showed that the anticancer activity of *A. muricata* fruit juice against HeLa cells had an IC_50_ value of 252.33 ppm. In contrast, Suyatmi *et al*. [52] stated that the anticancer activity of the ethanol extract of *A. muricata* leaves against HeLa cells (cervical) had an LC_50_ value of 97 ppm. A previous study by Artanti *et al*. [53] showed that the cervical anticancer activity of polyketide derivatives from the leaf extract of *A. muricata* was 77.09 ppm.

In the present study, the five compounds identified were (6S, 7aR)-6-hydroxy-4,4,7a-trimethyl-6,7-dihydro-5H-1-benzofuran-2-one or loliolide (Compound 1), cocamidopropyl betaine (Compound 2), N-[3-(dimethylamino)propyl]dodecanamide or lauramidopropyl dimethylamine (Compound 3), linolenic acid (Compound 4), and 1-dodecyl-2-azepanone or laurocapram (Compound 5). Based on the findings of previous studies, only two compounds have anticancer activity, namely, Compounds 1 and 4. However, no reports had indicated the other three compounds. This finding is consistent with the study by Susanti *et a*l. [54], who stated that the methanol extract of harp (*Sandoricum koetjape*) leaves containing loliolide compounds had an anticancer activity against P-388 murine leukemia cells. Similarly, Grabarczyk *et al*. [55] stated that loliolide compounds extracted from the *Opisthobranch* genus were able to inhibit the growth of human nasopharyngeal carcinoma KB (tumor cells of human nasopharyngeal carcinoma KB) and P-388 murine lymphocytic leukemia tumor cells. Loliolide and epi-loliolide compounds have inhibitory activities on the growth of human hepatocarcinoma (HepG-2) cells [56]. Furthermore, Elsayed *et al*. [57] stated that a mixture of fatty acids containing linolenic acid from Egyptian beebread has antibacterial activities against *Staphylococcus aureus*, *Escherichia coli*, *Bacillus subtilis*, and PC-3 as well as HepG-2 cancer cells. The toxicity of g-linolenic acid to HA-109A mouse hematoma cells (rat hematoma HA-109A cell) below 5 ppm was reported by Hayashi *et al*. [58].

## Conclusion

Based on the findings of this study, the cervical anticancer activity of the *A. squamosa* L. leaf isolate positively inhibited the growth of the HeLa cells with an IC_50_ value of 70.9021 ppm. Furthermore, a total of five compounds were detected in the isolate, namely, (6*S*, 7*aR*)-6-hydroxy-4,4,7*a*-trimethyl-6,7-dihydro-5*H*-1-benzofuran-2-one or loliolide, cocamidopropyl betaine, N-[3-(dimethylamino)propyl]dodecanamide or lauramidopropyl dimethylamine, linolenic acid, and 1-dodecyl-2-azepanon or laurocapram.

## Authors’ Contributions

MDS: Designed the study, collected the samples, performed laboratory work, analyzed the data, and wrote the manuscript. WSR: Designed the study, collected the samples, performed laboratory work, analyzed the data, and wrote the manuscript. MAD: Analyzed the data, and wrote the manuscript. KKA: Designed the study, analyzed the data, and wrote the manuscript. All authors read and approved the final manuscript.

## References

[1] Huang C.Y, Ju D.T, Chang C.F, Reddy P.M, Velmurugan B.K (2017). A review on the effects of current chemotherapy drugs and natural agents in treating non-small cell lung cancer. Biomedicine (*Taipei*).

[2] Siddiqui M, Rajkumar S.V (2012). The high cost of cancer drugs and what we can do about it. Mayo Clin. Proc.

[3] Cragg G.M, Newman D.J (2013). Natural products:A continuing source of novel drug leads. Biochim. Biophys. Acta.

[4] Dias D.A, Urban S, Roessner U (2012). A historical overview of natural products in drug discovery. Metabolites.

[5] Sharma G.N, Dave R, Sanadya J, Sharma P, Sharma K.K (2010). Various types and management of breast cancer:An overview. J. Adv. Pharm. Technol. Res.

[6] Atanasov A.G, Waltenberger B, Pferschy-Wenzig E.M, Linder T, Wawrosch C, Uhrin P, Temml V, Wang L, Schwaiger S, Heiss E.H, Rollinger J.M, Schuster D, Breuss J.M, Bochkov V, Mihovilovic M.D, Kopp B, Bauer R, Dirsch V.M, Stuppner H (2015). Discovery and resupply of pharmacologically active plant derived natural products:A review. Biotechnol. Adv.

[7] Zahid M, Mujahid M, Singh P.K, Farooqui S, Singh K, Parveen S, Arif M (2018). *Annona squamosa* Linn. (custard apple):An aromatic medicinal plant fruit with immense nutraceutical and therapeutic potentials. Int. J. Pharm. Sci. Res.

[8] Chavan M.J, Wakte P.S, Shinde D.B (2010). Analgesic and anti-inflammatory activity of caryophyllene oxide from *Annona squamosa* L. bark. *Phytomedicine*.

[9] Kalidindi N, Thimmaiah N.V, Jagadeesh N.V, Nandeep R, Swetha S, Kalidindi B (2015). Antifungal and antioxidant activities of organic and aqueous extracts of *Annona squamosa* Linn. leaves. J. Food Drug Anal.

[10] Bhattacharya A, Chakraverty R (2016). The pharmacological properties of *Annona squamosa* Linn:A review. Int. J. Pharm Eng.

[11] Singh Y, Bhatnagar P, Thakur N (2019). A review on insight of immense nutraceutical and medicinal potential of custard apple (*Annona squamosa* Linn.). Int. J. Chem. Stud.

[12] Ma C, Chen Y, Chen J, Li X, Chen Y (2017). A review on *Annona squamosa* L.: Phytochemicals and biological activities. Am. J. Chin. Med.

[13] Pardhasaradhi B.V.V, Reddy M, Ali A.M, Kumari A.L, Khar A (2004). Antitumour activity of *Annona squamosa* seed extracts is through the generation of free radicals and induction of apoptosis. Indian J. Biochem. Biophys.

[14] Pandey N, Barve D (2011). Phytochemical and pharmacological review on *Annona squamosa* Linn. Int. J. Res. Pharm. Biomed. Sci.

[15] Biba V.S, Amily A, Sangeetha S, Remani P (2014). Anticancer, antioxidant and antimicrobial activity of Annonaceae family. World J. Pharm. Pharm. Sci.

[16] Wang D.S, Rizwani G.H, Guo H, Ahmed M, Ahmed M, Hassan S.Z, Hassan A, Chen Z.S, Xu R.H (2014). *Annona squamosa* Linn:Cytotoxic activity found in leaf extract against human tumor cell lines. Pak. J. Pharm. Sci.

[17] Sumithra P, Gricilda S.F, Vimala G, Sathya J, Sankar V, Saraswathi R (2014). Anticancer activity of *Annona squamosa* and *Manilkara zapota* flower extract against MCF-7 cell line. Pharm. Sin.

[18] Veerakumar S, Amanulla S. S. D, Ramanathan K (2016). Anticancer efficacy of ethanolic extracts from various parts of *Annona squamosa* on MCF-7 cell line. J. Pharmacogn. Phyther.

[19] Oo W.M, Khine M.M (2017). Pharmacological activities of *Annona squamosa*:Updated review. Int. J. Pharm. Chem.

[20] Vanitha V, Hemalatha S, Pushpabharathi N, Amudha P, Jayalakshmi M (2017). Fabrication of nanoparticles using *Annona squamosa* leaf and assessment of its effect on liver (Hep G2) cancer cell line. IOP Conf. Ser. Mater Sci. Eng.

[21] Sandeep, Mittal A (2017). *Annona squamosa*:Seetaphal:A review. Int. J. Compr. Adv. Pharmacol.

[22] Al-Ghazzawi A.M (2019). Anticancer activity of new benzylisoquinoline alkaloid from Saudi plant *Annona squamosa*. BMC Chem.

[23] Fadholly A, Proboningrat A, Iskandar R.D, Rantam F, Sudjarwo S (2019). *In vitro* anticancer activity *Annona squamosa* extract nanoparticle on WiDr cells. J. Adv. Pharm. Technol. Res.

[24] Makbruri Saleh I, Hidayat R (2019). The anticancer activity of Srikaya leaves fraction (*Annona squamosa* L.):An *in vitro* study. Biosci. Med.

[25] Nugraha A.S, Damayanti Y.D, Wangchuk P, Keller P.A (2019). Anti-infective and anti-cancer properties of the Annona species:Their ethnomedicinal uses, alkaloid diversity, and pharmacological activities. Molecules.

[26] Shehata M.G, Abu-Serie M.M, Abd El-Aziz N.M, El-Sohaimy S.A (2021). Nutritional, phytochemical, and *in vitro* anticancer potential of sugar apple (*Annona squamosa*) fruits. Sci. Rep.

[27] El-Darier S.M, Abdelhady E.F (2017). Phytochemistry and cytotoxic activity of *Annona squamosa* L. fruit pulp against human carcinoma cell lines. Cancer Biol.

[28] Mehta S.D, Paliwal S (2018). Phytochemical analysis, liquid chromatography, and mass spectroscopy and *in vitro* anticancer activity of *Annona squamosa* seeds Linn. Asian J. Pharm. Clin. Res.

[29] Mclaughlin J.L, Rogers L.L, Anderson J.E (1998). The use of biological assays to evaluate botanicals. Ther. Innov. Regul. Sci.

[30] Silva T.M.S, Nascimento R.J.B, Batista M.M, Agra M.F, Camara C.A (2007). Brine shrimp bioassay of some species of *Solanum* from Northeastern Brazil. Rev. Bras. Farmacogn.

[31] Meyer B.N, Ferrigni N.R, Putnam J.E, Jacobsen L.B, Nicholas D.E, Mclaughlin J.L (1982). Brine shrimp:A convenient general bioassay for active plant constituents. J. Med. Plant Res.

[32] Anderson J.E, Goetz C.M, McLaughlin J.L, Suffness M (1991). A blind comparison of simple bench-top bioassays and human tumour cell cytotoxicities as antitumor prescreens. Phytochem. Anal.

[33] Sadino A, Sahidin I, Wahyuni W (2017). Acute toxicity of ethanol extract of *Polygonum pulchrum* blume using brine shrimp lethality test method. Pharmacol. Clin. Pharm. Res.

[34] Scherer W.F, Syverton J.T, Gey G.O (1953). Studies on the propagation *in vitro* of poliomyelitis viruses:IV. Viral multiplication in a stable strain of human malignant epithelial cells (strain HeLa) derived from an epidermoid carcinoma of the cervix. J. Exp. Med.

[35] Rahbari R, Sheahan T, Modes V, Collier P, Macfarlane C (2009). A novel L1 retrotransposon marker for HeLa cell line identification. Biotechniques.

[36] Corporation W (2014). Liquid Chromatography/Mass spectrometry Manual.

[37] The Association of Official Analytical Chemists (1984) Official Methods of Analysis.

[38] Swantara I.M.D, Rita W.S, Suartha I.N (2018). Anticancer activities of toxic extract of *Xestospongia testudinaria* sponge from Sanur Beach, Bali, Indonesia. Res. J. Pharm. Biol. Chem. Sci.

[39] Swantara M.D, Rita W.S, Suartha N, Agustina K.K (2019). Anticancer activities of toxic isolate of *Xestospongia testudinaria* sponge. Vet. World.

[40] Colegate S.M, Molyneux R.J (1993). Bioactive Natural Products:Detection, Isolation and Structural Determination.

[41] Carballo J.L, Hernández-Inda Z.L, Pérez P, García-Grávalos M.D (2002). A comparison between two brine shrimp assays to detect *in vitro* cytotoxicity in marine natural products. BMC Biotechnol.

[42] McCauley J, Zivanovic A, Skropeta D (2013). Bioassays for anticancer activities. Methods Mol. Biol.

[43] van Meerloo J, Kaspers G. J. L, Cloos J (2011). Cell sensitivity assays:The MTT assay. Methods Mol. Biol.

[44] Mosmann T (1983). Rapid colorimetric assay for cellular growth and survival:Application to proliferation and cytotoxicity assays. J. Immunol. Methods.

[45] Doyle A, Griffiths J.B (2000). Cell and Tissue Culture for Medical Research.

[46] Abdel-Hameed E.S.S, Bazaid S.A, Shohayeb M.M, El-Sayed M.M, El-Wakil E.A (2012). Cytotoxic activities of ethanolic and dichloromethane extract of leaves, stems, and flowers of Jarong [*Stachytarpheta jamaicensis* (L.) Vahl.] On HeLa and T47D cancer cell line. Eur. J. Med. Plants.

[47] Cante R.C, Garella I, Gallo M, Nigro R (2021). Effect of moisture content on the extraction rate of coffee oil from spent coffee grounds using Norflurane as solvent. Chem. Eng. Res. Des.

[48] Alimzhanova M.B, Kenessov B.N, Nauryzbayev M.K, Koziel J.A (2012). Effects of moisture content and solvent additive on headspace solid-phase microextraction of total petroleum hydrocarbons from soil. Eur. Chem. J.

[49] Swantara I.M.D, Bawa I.G.A, Suprapta D.N, Agustina K.K, Temaja I.G.R (2020). Identification of *Michelia alba* barks extract using gas chromatography-mass spectrometry (GC-MS) and its antifungal properties to inhibit microbial growth. Biodiversitas.

[50] Yajid A.I, Ab Rahman H.S, Wong M.P.K, Wan Zain W.Z (2018). Potential benefits of *Annona muricata* in combating cancer:A review. Malays. J. Med. Sci.

[51] Widyanto R.M, Putri R.M.R, Kurniasari F.N, Yunimar Y, Utomo B (2020). Free radical scavenging and cytotoxic assay of soursop fruit juice (*Annona muricata* Linn.). on cervical cancer cell lines (HeLa). J. Gizi Diet Indones.

[52] Suyatmi S, Suselo Y.H, Jusuf S.A (2012). The selective cytotoxicity of ethanolic extract of *Annona muricata* leaf on HeLa cervical cancer cells. In:Research and Application on Traditional Complementary and Alternative Medicine in Health Care. Surakarta, Indonesia.

[53] Artanti N.A, Astirin O.P, Prayito A, Widiyaningsih R.F, Prihapsara F (2016). Polyketide Derivatives from *Annona muricata* Linn. Leaves as Potential Anticancer Material by Combination Treatment with Doxorubicin on Hela Cell Line. International Conference on Advanced Materials for Better Future.

[54] Susanti F.E, Sugita P, Ambarsari L (2016). Purification of active compounds from kecapi leaves that have potential as anticancer for *in vitro* on murine cells leukemia P-388. Int. J. Chem. Sci.

[55] Grabarczyk M, Wińska K, Mączka W, Potaniec B, Anioł M (2015). Loliolide the most ubiquitous lactone. Folia Biol. Oecol.

[56] Gangadhar K.N, Rodrigues M.J, Pereira H, Gaspar H, Malcata F.X, Barreira L, Varela J (2020). Anti-hepatocellular carcinoma (HepG2) activities of monoterpene hydroxy lactones isolated from the marine microalga *Tisochrysis lutea*. Mar. Drugs.

[57] Elsayed N, El-Din H.S, Altemimi A.B, Ahmed H.Y, Pratap-Singh A, Abedelmaksoud T.G (2021). *In vitro* antimicrobial, antioxidant and anticancer activities of Egyptian citrus beebread. Molecules.

[58] Hayashi Y, Fukushima S, Hirata T, Kishimoto S, Katsuki T, Nakano M (1990). Anticancer activity of free g-linolenic acid on AH-109A rat hepatoma cells and the effect of serum albumin on anticancer activity of g-linolenic acid *in vitro*. J. Pharmacobiodyn.

